# Asymptomatic and Submicroscopic Carriage of *Plasmodium knowlesi* Malaria in Household and Community Members of Clinical Cases in Sabah, Malaysia

**DOI:** 10.1093/infdis/jiv475

**Published:** 2015-10-03

**Authors:** Kimberly M. Fornace, Nor Afizah Nuin, Martha Betson, Matthew J. Grigg, Timothy William, Nicholas M. Anstey, Tsin W. Yeo, Jonathan Cox, Lau Tiek Ying, Chris J. Drakeley

**Affiliations:** 1 London School of Hygiene and Tropical Medicine; 2 Royal Veterinary College, London, United Kingdom; 3 Biotechnology Research Institute, Universiti Sabah Malaysia; 4 Infectious Diseases Society Sabah–Menzies School of Health Research Clinical Research Unit; 5 Sabah Department of Health, Clinical Research Centre; 6 Jesselton Medical Centre, Kota Kinabalu, Malaysia; 7 Menzies School of Health Research, Darwin, Australia

**Keywords:** malaria, *Plasmodium knowlesi*, submicroscopic infection, asymptomatic parasitemia

## Abstract

Although asymptomatic carriage of human malaria species has been widely reported, the extent of asymptomatic, submicroscopic *Plasmodium knowlesi* parasitemia is unknown. In this study, samples were obtained from individuals residing in households or villages of symptomatic malaria cases with the aim of detecting submicroscopic *P. knowlesi* in this population. Four published molecular assays were used to confirm the presence of *P. knowlesi.* Latent class analysis revealed that the estimated proportion of asymptomatic individuals was 6.9% (95% confidence interval, 5.6%–8.4%). This study confirms the presence of a substantial number of asymptomatic monoinfections across all age groups; further work is needed to estimate prevalence in the wider community.

Malaria epidemiological surveys have reported a substantial proportion of individuals with low-density infections that are not detectable by conventional microscopy [1]. Meta-analysis of studies that used molecular amplification techniques suggest that these submicroscopic infections represent on average 50% of malaria parasite infections. This proportion can be as high as 80% in areas of malaria transmission where the community parasite prevalence detected by microscopy is <10% [2].

Although submicroscopic carriage of human malaria parasite species, particularly *Plasmodium falciparum* and *Plasmodium vivax*, has been described, limited data about such carriage are available for zoonotic malaria species. *P. knowlesi*, a zoonotic malaria parasite maintained by macaques, has been described throughout Southeast Asia and is now the most common cause of human malaria in Malaysian Borneo [3]. To our knowledge, limited asymptomatic *P. knowlesi* infection has only been identified in 2 studies, both of which were performed in Vietnam, and submicroscopic parasitemia in people has not been widely reported. During 2 cross-sectional malariometric surveys in Vietnam, 3 individuals were identified by molecular techniques as *P. knowlesi* positive [4]. These infections were found in 2 young children (age, 2 and 3 years) and a man (age, 27 years), all of whom were asymptomatic at the time and for 6 months following the survey. Similarly, multiple coinfections with *P. knowlesi* were detected in younger age groups through active case detection [5].

Despite rarely causing clinical disease, submicroscopic malaria infections can contribute to malaria transmission. Experimental evidence has demonstrated that individuals with submicroscopic infections are capable of infecting mosquitoes; while these individuals may infect fewer mosquitoes than individuals with higher parasite counts, the high numbers of individuals with low-density infections may lead them to contribute substantially to malaria transmission [6]. Understanding the prevalence of these infections and the extent to which they contribute to malaria transmission is critical for designing effective malaria control programs.

Data on submicroscopic parasitemia are also needed to better understand disease progression. Both parasite and host factors will influence whether infections remain asymptomatic or become symptomatic and potentially life threatening. The data on *P. knowlesi* infection dynamics in exposed populations are very limited. The parasite has a distinct 24-hour asexual development cycle, with common-severe-disease and case-fatality rates similar to those recorded for *P. falciparum* [7]. This potential for rapid disease progression makes improving our understanding of *P. knowlesi* particularly important.

This study aimed to detect potential asymptomatic *P. knowlesi* cases by screening individuals residing in the same households and villages of clinical *P. knowlesi* cases recruited during a population-based case control study in an area of known *P. knowlesi* transmission in Northwestern Sabah, Malaysia [8]. *P. knowlesi* is the main cause of clinical human malaria in this region and clustering of cases at household level has previously been reported [9]. Individuals were screened by microscopy and multiple molecular methods to determine whether asymptomatic *P. knowlesi* carriage is present in this population and to estimate the proportion of infected individuals.

## METHODS

### Study Design

The study sites in Kudat and Kota Marudu districts, Sabah, Malaysia, have been described elsewhere [8]. The area is served by 2 district hospitals and numerous referral clinics. Malaria is a notifiable disease in Malaysia, and all patients with malaria have access to free treatment.

As part of a case control study, consenting clinical cases positive for any species of malaria by microscopy were recruited at district hospitals and visited at their homes within 2 weeks of initial infection detection. Community controls were randomly selected afebrile individuals residing in the same village as malaria cases for the previous 3 weeks, as described elsewhere [8]. Blood samples were also collected from all consenting individuals residing in the same household as both cases and controls; these included a specimen obtained for a blood smear to detect malaria parasites by microscopy and whole-blood specimens stored on filter paper (3 MM; Whatman, Maidstone, United Kingdom) and collected in a 500-µL tube containing ethylenediaminetetraacetic acid (Becton-Dickinson, Franklin Lakes, New Jersey). Demographic details were recorded for all individuals residing in the same households or villages as cases, and individuals were asked about their history of fever. Malaysian health policy mandates that all cases of malaria are referred to the district hospital for treatment, and the case-control study prospective surveillance system [8] enabled detection of subsequent clinical disease among asymptomatically infected controls.

### Ethics Approval

This study was approved by the Medical Research Sub-Committee of the Malaysian Ministry of Health and by the research ethics committees of the London School of Hygiene and Tropical Medicine and the Menzies School of Health Research (NMRR-12-537-12568). Written informed consent was obtained from all participants in this study.

### Detection of Malaria Parasite Infection

Thick and thin blood smears were examined by a trained malaria microscopist. DNA was extracted from 10-µL red blood cell pellets by using the Chelex-100 boiling method, and a nested polymerase chain reaction (PCR) assay targeting the *Plasmodium* small subunit ribosomal RNA (ssRNA) gene was performed to identify *Plasmodium* genus–positive samples as described elsewhere [10] and in the Supplementary Materials. Primers targeting a region of the ssRNA product of the first nested PCR were then used to detect *P. knowlesi* [11]. Positive controls of confirmed clinical cases of *P. knowlesi* and other species were used for all PCR reactions.

Owing to difficulties in determining species in some of the *Plasmodium* genus–positive samples, additional methods were performed on a subset of 374 samples. A nested PCR assay targeting the cytochrome B gene of *P. knowlesi* was used to identify *P. knowlesi–*positive individuals [12]. Samples were also evaluated using 2 real-time PCR assays, one targeting the *P. knowlesi* ssRNA gene and the other targeting the *P. knowlesi* strain H chromosome 13 plasmepsin gene [13, 14], as described in the Supplementary Materials.

### Statistical Analysis

Data were analyzed using R statistical software, version 3.1.1 (R Foundation for Statistical Computing, Vienna, Austria; available at: http://www.R-project.org). In the absence of a gold standard for *P. knowlesi* species–specific diagnosis, latent class analysis was used to estimate the proportion of infected individuals and the sensitivity and specificity of these tests, using the randomLCA package in R (v1.0.2) [15]. Test type was included as a random effect to account for conditional dependence between assays. Competing models with 1–3 latent classes, representing possible diagnostic classes, were developed, and model selection was based on the Bayesian information criterion. Posterior probabilities for each latent class were estimated, using parametric bootstrap methods to estimate confidence intervals (CIs). Individuals were assigned to the infected or uninfected classes on the basis of predicted probabilities.

## RESULTS

A total of 1147 blood samples were collected from December 2012 through May 2014. Only 1 individual was microscopy positive but did not report a history of fever. Initial *Plasmodium* genus-specific PCR results, obtaining using the ssRNA primers as described previously [10], found that 18% of these individuals (206 of 1147) were positive for *Plasmodium* species. From this sample set, 1.7% (20 of 1147; 95% CI, 1.0%–2.5%) were confirmed to be *P. knowlesi* positive, using the *P. knowlesi*–specific ssRNA primers [11]. Because *P. knowlesi* is the predominant cause of human malaria in this area and many *Plasmodium* genus–positive samples could not be identified to the species level, alternate assays were performed on all 206 genus-positive samples and a subset of 168 genus-negative samples [12–14].

The genus-specific nested PCR was highly sensitive (sensitivity, 100%; 95% CI, 95%–100%), detecting all *P. knowlesi* infections identified by any other method. A total of 9.8% of samples (112 of 1147) had at least 1 positive *P. knowlesi* test result; of these 112, 26.8% (30) had 2 positive results, and 30.3% (34) had ≥3 positive test results. The diagnostic sensitivity and specificity of each *P. knowlesi–*specific assay for this population was estimated using latent class analysis (Table 1), demonstrating that the standard nested PCR used for *P. knowlesi* identification had a lower sensitivity to detect submicroscopic infections (estimated sensitivity, 15%; 95% CI, 8%–23%) than the other molecular assays used. Using these estimates of sensitivity and specificity, the infection prevalence in this population was estimated at 6.9% (95% CI, 5.6%–8.4%).

**Table 1.** Prevalence of Latent Classes and Conditional Probabilities for Latent Class Model

Assay, Result	No.^a^	Latent Class, Conditional Probability, %	
		Infected	Noninfected
ssRNA nested PCR			
Positive	20	15.0	2.7
Negative	1127	85.0	97.3
cytB nested PCR			
Positive	67	59.1	6.8
Negative	305	40.9	93.2
ssRNA real-time PCR			
Positive	71	87.9	2.4
Negative	264	12.1	97.6
Plasmepsin real-time PCR			
Positive	60	81.3	0.9
Negative	229	18.7	99.1

Latent classes were assigned using the results from all available assays.

Abbreviations: cytB, cytochrome B; PCR, polymerase chain reaction; ssRNA, small subunit RNA.

^a^ Some assays were not performed on all samples, owing to insufficient DNA availability.

The majority (87%; 95% CI, 79.8%–94.2%) of *P. knowlesi*–infected individuals predicted by the latent class analysis did not report a history of a fever (Table 2), similar to the noninfected population (947 of 1063 [89%]), and none of these individuals presented to the hospital with clinical malaria following this survey. Infected individuals were present in households of both cases and controls and in all age groups (Table 2). Households with multiple infected individuals were identified, including 11 households with 2 asymptomatic infected individuals and 2 households with 3 infected individuals. All but 1 of these households also reported a symptomatic case. The majority of infections with *P. knowlesi* were monoinfections; 5% (4) were coinfections with *P. falciparum*, and 2% (2) were coinfections with *P. malariae*.

**Table 2.** Demographic and Clinical Characteristics and Infection Prevalence Based on Probabilities of Latent Classes

Characteristic	Study Population, % (No.) (n = 1147)	*P. knowlesi* Infection, %^a^ (95% CI)
District		
Kota Marudu	39.8 (457)	9.0 (6.4–11.6)
Kudat	60.1 (690)	6.2 (4.4–8.0)
Residence		
Case household	44.6 (512)	8.4 (6.0–10.8)
Control household	55.4 (635)	6.5 (4.6–8.4)
Sex		
Male	46.6 (535)	6.2 (4.2–8.2)
Female	53.4 (612)	8.3 (6.1–10.5)
Age, y		
<15	28.2 (323)	9.6 (6.4–12.8)
15–45	42.9 (492)	6.7 (4.5–8.9)
46–60	16.9 (194)	6.7 (8.3–17.7)
>60	12.0 (138)	5.1 (1.4–8.8)
Self-reported fever in past month		
Yes	11.1 (127)	8.7 (3.8–13.6)
No	87.3 (1001)	7.3 (5.7–8.9)
Don't know	1.6 (19)	0
Microscopy result		
Positive	0.1 (1)	100
Negative	99.9 (1147)	7.2 (5.7–8.7)

Abbreviation: CI, confidence interval.

^a^ Based on probability of class membership (uninfected, median *P* = .998; infected, median *P* = .997).

## DISCUSSION

This is the first study to describe a high level of submicroscopic, asymptomatic *P. knowlesi* carriage in an exposed human population. Although this is not a true prevalence survey and many of the infections detected were from individuals residing in the same household as symptomatic cases, infections were also found in unrelated individuals residing in the same village during that time. Moreover, a high proportion of infections were detected in children aged <15 years and women, groups not previously considered to be at high risk for *P. knowlesi* infection. Although a small proportion of infected individuals reported a history of fever, none had infections that were reported to develop into symptomatic malaria. However, longitudinal studies are needed to fully understand the disease progression and potential development of acute disease. Given the ongoing case recruitment in these health facilities [8], the mandatory referral policy, and the previous history of members of these households presenting to the clinic, it is unlikely that these infections developed into clinical malaria, suggesting that there is a substantial number of asymptomatic *P. knowlesi* infections in the community. Further, the distribution of these infections in different demographic groups may not be captured by the passive health surveillance systems.

The majority of infections were submicroscopic and could not be detected by conventional malaria parasite microscopy. Unlike previously reported submicroscopic infections, the majority (93%) of infections were not associated with coinfection involving other species. Additionally, the inconsistent results obtained from multiple published and validated molecular assays demonstrate that the parasites causing these infections may be at or below the level of detection for assays developed for use primarily on human clinical samples. Analysis of relatively small volumes of blood collected from individuals with very-low-density infections means that parasites present in such samples may be missed even by repeat PCR assays. Understanding the wider community prevalence will require better-optimized molecular assays, as well as population-level surveys.

It remains unknown whether and how much humans contribute to the infectious reservoir for *P. knowlesi* transmission. While the submicroscopic parasite densities of other malaria parasite species have been shown to be capable of infecting mosquitoes, all experimental infections with *P. knowlesi* have involved specimens collected from clinical malaria cases [3]. The identification of multiple human infections in different demographic groups within limited geographical areas, including households, suggests the possibility of peri-domestic transmission. Although this cannot be directly attributed to human-to-human transmission, it is probably indicative of exposure to the same infected vectors. Further studies could use molecular typing techniques to identify whether strains of *P. knowlesi* identified within the same areas are likely to have a common source. Entomological and primatological studies are also needed to evaluate the presence of potential *P. knowlesi* vectors in these village environments and the proximity of infected reservoirs.

Because this study included only individuals in the same household or village as that of a symptomatic case, these data cannot be used to generalize about the community prevalence of *P. knowlesi* or to further understand spatial and temporal patterns of *P. knowlesi* infection. Similarly, because samples were collected at a single time point for each individual, the duration and fluctuations in parasite densities over time cannot be determined. Longitudinal data, as well as data on treatment-seeking behaviors, for different demographic groups are required to determine factors contributing to whether specific groups are underrepresented by hospital surveillance systems.

Despite these limitations, this study illustrates the presence of asymptomatic *P. knowlesi* monoinfections within communities located in *P. knowlesi*–endemic areas and highlights the need for further studies to evaluate population-wide prevalence. Current molecular tools are still limited for detection of parasites present at very low densities, and these need further evaluation and optimization. Additional community-based surveys are currently planned to evaluate the prevalence of these infections in the wider community.

## Supplementary Material

Supplementary Data
