# Supplementary material for: Asymptomatic and Submicroscopic Carriage of Plasmodium knowlesi Malaria in Household and Community Members of Clinical Cases in Sabah, Malaysia
Source: J Infect Dis. 2015 Oct 3;213(5):784–7. doi: 10.1093/infdis/jiv475 (PMC4747612; doi:10.1093/infdis/jiv475)
Supplement: Supplementary Data [file supp_213_5_784__index.html]

Asymptomatic and submicroscopic carriage of Plasmodium knowlesi malaria in household and community members of clinical cases in Sabah, Malaysia — Asymptomatic and Submicroscopic Carriage of Plasmodium knowlesi Malaria in Household and Community Members of Clinical Cases in Sabah, Malaysia — Asymptomatic and Submicroscopic Carriage of Plasmodium knowlesi Malaria in Household and Community Members of Clinical Cases in Sabah, Malaysia — Supplementary Data 

# Asymptomatic and Submicroscopic Carriage of *Plasmodium knowlesi* Malaria in Household and Community Members of Clinical Cases in Sabah, Malaysia

## Supplementary Data

Supplementary Data

- Supplementary Data - Docx file
