# Supplementary material for: Asymptomatic and Submicroscopic Carriage of Plasmodium knowlesi Malaria in Household and Community Members of Clinical Cases in Sabah, Malaysia
Source: J Infect Dis. 2015 Oct 3;213(5):784–7. doi: 10.1093/infdis/jiv475 (PMC4747612; doi:10.1093/infdis/jiv475)
Supplement: Supplementary Data [file supp_jiv475_jiv475supp.docx]

**Supplementary information:**

*Molecular detection of Plasmodium:*

Multiple molecular methods were used to identify *P. knowlesi* infections. DNA was extracted from 10µl red blood cell pellets using the Chelex-100 boiling method. Initial screening for *Plasmodium* infections was done using a nested PCR assay targeting the ssRNA [1]. This nested PCR assay used the genus-specific primers rPLU1 (5’-TCA AAG ATT AAG CCA TGC AAG TGA-3’) and rPLU5 (5’-CCT GTT GTT GCC TTA AAC TTC-3’) for nest 1 using 4 μl of DNA and rPLU3 (5’-TTT TTA TAA GGA TAA CTA CGG AAA AGC TGT-3’) and rPLU4 (5’-TAC CCG TCA TAG CCA TGT TAG GCC AAT ACC-3’) for nest 2 using 2 μl of template [1]. Thermal cycling conditions for primary and nested PCRs were 35 cycles at 94°C, 60°C and 72°C. Products were visualised on a 2% agarose gel. The sensitivity of this assay as a screening tool was evaluated by screening all genus positive samples and a subset of 180 genus negative samples.

*Detection of Plasmodium species:*

Species specific primers targeting the ssRNA gene were used as described by [1], using the same conditions as nest 1. As cross reactivity between *P. vivax* and *P. knowlesi* has been reported, primers targeting an alternate region of the nest 1 ssRNA product were used for *P. knowlesi*; these comprised PkF1140 (5’-GATTCATCTATTAAAAATTTGCTTC-3’) and PkR1150 (5’- TCTTTTCTCTCCGGAGATTAGAACTC-3’) [2]. Conditions for nest 2 of this PCR were 35 cycles at 50°C, 72°C and 94°C using 2μl of template DNA and all results were visualised on a 2% agarose gel. Positive controls for confirmed clinical cases of *P. knowlesi* and other species were used for all PCR reactions.

Due to difficulties in identifying some of the genus positive samples to species, additional methods were used on a subset of 374 samples, including 168 genus negative samples and 206 genus positive samples. Previously reported minimum detection thresholds are included in Supplementary Table 1.

Supplementary Table 1: Minimum detection thresholds of *P. knowlesi* assays

| Assay | Study population |  |
| --- | --- | --- |
| ssRNA Nested PCR | 1 to 10 parasite genomes per reaction [2] | |
| CytB Nested PCR | Not reported [3] | |
| ssRNA Real Time PCR | 10 gene copies per reaction [4] | |
| Plasmepsin Real Time PCR | 10 to 100 gene copies per μl [5], 1-6 parasites per μl [6] | |

A nested PCR assay targeting the cytochrome B gene of *P. knowlesi* was used to identify *P. knowlesi* positives [3]. The primers PCBF (5’-ATGCTTTATTATGGATTGGATGTC-3’) and PCBRed (5’-ACATAATTATAACCTTACGGTCTG-3’) were used for the first nest and PkCB (5’-TATTCTTCTTTAGTGGATTATTTA-3’) and PkCBed (5’-GTATTGTTCTAATCAGTGTA-3’) were used for the second nest [7, 8]. Thermocycler conditions were 95°C, 50°C, 72°C, with 35 cycles for the first nest and 25 cycles for the second nest.

Samples were also run using two real-time PCR assays, one targeting the *P. knowlesi* ssRNA gene and one targeting strain H chromosome 13 plasmepsin [4, 5]. For the ssRNA real-time assay, the primers Plasmo 1 [5’-GTTAAGGGAGTGAAGACGATCAGA-3’] and Plasmo 2 [5’AACCCAAAGACTTTGATTTC TCATAA-3’], and a FAM-labelled Pk probe [5’-FAM-CTCTCCGGAGATTAGAACTCTTAGATTGCT -BHQ1-3’] were employed [6]. Real-time PCR reactions contained 200 nM of each primer, 80 nM probe, SensiFAST™ Probe No-ROX mastermix (Bioline, London, UK) and 2 μl of DNA template in a total volume of 20 μl. Reactions were performed using the CFX96 real-time PCR detection system (Biorad, Hemel Hempstead, UK) under the following cycling conditions: initial denaturation at 95°C for 5 min followed by 45 cycles of 95°C for 10 sec and 60°C for 30 sec. For the plasmepsin real-time assay, the forward and reverse primer sequences were 5’- TAACATGGTAATCATACATAAGG-3’ and 5’-TAAGGAAATGCCAACTCTTG-3’, respectively, and the probe was FAM -TCAGCCAACAACACTTACAG-BHQ1 [7]. Reaction conditions were as for the ssRNA assay except the probe was used at 200 nM. The cycling conditions were: initial denaturation at 95°C for 5 min followed by 40 cycles of 95°C for 10 sec and 55°C for 30 sec. Ct values of 45 and 40 were used for the ssRNA and plasmepsin assays respectively.

1. Singh, B., et al., *A genus- and species-specific nested polymerase chain reaction malaria detection assay for epidemiologic studies.* Am J Trop Med Hyg, 1999. **60**(4): p. 687-92.

2. Imwong, M., et al., *Spurious amplification of a Plasmodium vivax small-subunit RNA gene by use of primers currently used to detect P. knowlesi.* J Clin Microbiol, 2009. **47**(12): p. 4173-5.

3. Kawai, S., et al., *Detection of Plasmodium knowlesi DNA in the urine and faeces of a Japanese macaque (Macaca fuscata) over the course of an experimentally induced infection.* Malar J, 2014. **13**: p. 373.

4. Divis, P.C., et al., *A TaqMan real-time PCR assay for the detection and quantitation of Plasmodium knowlesi.* Malar J, 2010. **9**: p. 344.

5. Reller, M.E., et al., *Multiplex 5' nuclease quantitative real-time PCR for clinical diagnosis of malaria and species-level identification and epidemiologic evaluation of malaria-causing parasites, including Plasmodium knowlesi.* J Clin Microbiol, 2013. **51**(9): p. 2931-8.

6. Lau, Y.L., et al., *Comparison of three molecular methods for the detection and speciation of five human Plasmodium species.* Am J Trop Med Hyg, 2015. **92**(1): p. 28-33.

7. Putaporntip, C., P. Buppan, and S. Jongwutiwes, *Improved performance with saliva and urine as alternative DNA sources for malaria diagnosis by mitochondrial DNA-based PCR assays.* Clin Microbiol Infect, 2011. **17**(10): p. 1484-91.

8. Tanizaki, R., et al., *First case of Plasmodium knowlesi infection in a Japanese traveller returning from Malaysia.* Malar J, 2013. **12**: p. 128.
